# A new recombineering system for *Photorhabdus* and *Xenorhabdus*

**DOI:** 10.1093/nar/gku1336

**Published:** 2014-12-24

**Authors:** Jia Yin, Hongbo Zhu, Liqiu Xia, Xuezhi Ding, Thomas Hoffmann, Michael Hoffmann, Xiaoying Bian, Rolf Müller, Jun Fu, A. Francis Stewart, Youming Zhang

**Affiliations:** 1 Hunan Provincial Key Laboratory for Microbial Molecular Biology-State Key Laboratory Breeding Base of Microbial Molecular Biology, College of Life Science, Hunan Normal University, 410081 Changsha, People's Republic of China; 2 Shandong University–Helmholtz Institute of Biotechnology, State Key Laboratory of Microbial Technology, School of Life Science, Shandong University, Shanda Nanlu 27, 250100 Jinan, People's Republic of China; 3 Department of Genomics, Dresden University of Technology, BioInnovations-Zentrum, Tatzberg 47-51, 01307 Dresden, Germany; 4 Helmholtz Institute for Pharmaceutical Research, Helmholtz Centre for Infection Research and Department of Pharmaceutical Biotechnology, Saarland University, PO Box 151150, 66041 Saarbrücken, Germany; 5 Department of Protein Evolution, Max-Planck Institute for Developmental Biology, Spemannstr. 35, 72076 Tübingen, Germany

## Abstract

Precise and fluent genetic manipulation is still limited to only a few prokaryotes. Ideally the highly advanced technologies available in *Escherichia coli* could be broadly applied. Our efforts to apply *lambda* Red technology, widely termed ‘recombineering’, in *Photorhabdus* and *Xenorhabdus* yielded only limited success. Consequently we explored the properties of an endogenous *Photorhabdus luminescens lambda* Red-like operon, Plu2934/Plu2935/Plu2936. Bioinformatic and functional tests indicate that Plu2936 is a 5’-3’ exonuclease equivalent to Redα and Plu2935 is a single strand annealing protein equivalent to Redβ. Plu2934 dramatically enhanced recombineering efficiency. Results from bioinformatic analysis and recombineering assays suggest that Plu2934 may be functionally equivalent to Redγ, which inhibits the major endogenous *E. coli* nuclease, RecBCD. The recombineering utility of Plu2934/Plu2935/Plu2936 was demonstrated by engineering *Photorhabdus* and *Xenorhabdus* genomes, including the activation of the 49-kb non-ribosomal peptide synthase (NRPS) gene cluster *plu2670* by insertion of a tetracycline inducible promoter. After tetracycline induction, novel secondary metabolites were identified. Our work unlocks the potential for bioprospecting and functional genomics in the *Photorhabdus, Xenorhabdus* and related genomes.

## INTRODUCTION

Phage-encoded homologous recombination systems have greatly expanded recombinant DNA technology as well as providing accuracy and fluency in *Escherichia coli* genome engineering (1–6). Oligonucleotides and linear dsDNAs with homology arms as short as 35 nucleotides have been used to engineer high copy plasmids, large episomes and the *E. coli* chromosome with base pair precision. The required homology arms are short enough to be integrated into synthetic oligonucleotides, greatly expanding the utility of the technology, which is termed recombinogenic engineering or recombineering (7,8).

Two phage-encoded systems have been employed. The initial recombineering breakthrough was based on the rac phage RecE/RecT protein pair (1). However, we (2,3) and others (4–6) have mainly employed the equivalent *lambda* Red operon, which encodes Redα, Redβ and Redγ. Redα, like RecE, is a 5’ to 3’ exonuclease that generates 3’-ended ssDNA overhangs (9,10). Redβ, like RecT, is a single strand annealing protein (SSAP) that binds to ssDNA and forms a nucleoprotein filament with complementary ssDNA (11–15). Each pair, Redα/Redβ and RecE/RecT has a specific protein–protein interaction that promotes dsDNA homologous recombination (16).

Redγ is an inhibitor of the major *E. coli* exonuclease, RecBCD, which rapidly destroys linear dsDNA (17). The Redγ dimer is a DNA mimic that binds to and inhibits the exonuclease and helicase activities of RecBCD (18–22). The RecE/RecT operon does not appear to include an equivalent of Redγ, which may relate to the mechanistic difference that we identified recently (23). Nevertheless, inclusion of Redγ with RecE/RecT promotes homologous recombination through inhibition of RecBCD and thereby retention of dsDNA intermediates.

The phage *lambda* Red system can be directly used in some gram-negative bacteria. In addition to *E. coli*, recombineering has also been reported as workable in *Salmonella enterica* (24) and *Agrobacterium tumefaciens* (25). However in more distant species, host-specific phage-derived recombination systems are required. The new recombineering system has been established in *Yersinia pseudotuberculosis* (26), *Mycobacterium tuberculosis* (27,28), *Pseudomonas syringae* (29), *Lactococcus lactis* (30), and *Clostridium perfringens* (31).

*Photorhabdus* and *Xenorhabdus* are closely related genera according to 16s rRNA gene sequence analysis (32–34). They are symbiotic to soil nematodes belonging to the species *Heterorhabditis bacteriophora* and are highly pathogenic to insects (35,36). Genome projects of *Photorhabdus luminescens* and *Xenorhabdus stockiae* revealed many large biosynthetic gene clusters (37), which could prove useful as insecticides. Since *Photorhabdus* and *Xenorhabdus* are difficult to genetically manipulate, a recombineering system that overcomes these difficulties would be highly desirable. For example, a silent biosynthetic gene cluster could be activated by chromosomal modification to insert a promoter and thereby open an alternative way to prospect for useful bioactive compounds (38).

Here we report a recombineering system for *Photorhabdus* and *Xenorhabdus* based on three host-specific phage proteins from *P. luminescens*. Bioinformatics and recombineering tests indicate that Plu2935, Plu2936 and Plu2934 are functional analogs of Redβ, Redα and Redγ, respectively. Bioinformatic analysis suggests that Plu2934 may work like Redγ, which in *E. coli* inhibits the host exonuclease, RecBCD. Using these proteins, we efficiently modified the *Photorhabdus* and *Xenorhabdus* genomes. In particular, a 49-kb non-ribosomal peptide synthase (NRPS) gene cluster in *P. luminescens* (*plu2670)* was activated by placing a tetracycline inducible promoter in front of its start codon.

## MATERIALS AND METHODS

### Strains, plasmids and reagents

The wild-type bacterial strains and the mutants used are listed in Supplementary Table S1. The plasmids used are listed in Supplementary Table S2. All the expression plasmids used here to compare homologous recombination efficiencies are based on the pSC101 origin and the araC P_BAD_ promoter (39,40). The plasmids were constructed by recombineering either in GB08-red or in GB05-dir (23,41). When T4 DNA ligase was used, the DNA ligation products were dialyzed and then electroporated in *E. coli* GB2005. Genes encoding different Plu proteins were polymerase chain reaction (PCR) products amplified from *P. luminescens* genomic DNA and sequence-verified after cloning. Oligonucleotides were synthesized by Sigma-Genosys in Germany (Supplementary Table S3). Restriction enzymes, Phusion polymerase and DNA marker were supplied by New England Biolabs. The antibiotics were purchased from Invitrogen. *E. coli, P. luminescens* and *X. stockiae* were cultured in Luria–Bertani (LB) broth or on LB agar plates (1.2% agar) with ampicillin [*amp*] (100 μg ml^−1^), kanamycin [*kan*] (15 μg ml^−1^), chloramphenicol [*cm*] (15 μg ml^−1^) or gentamicin [*genta*] (5 μg ml^−1^) as required.

### Preparation of electrocompetent cells for recombineering

Various expression plasmids were electroporated into *E. coli, P. luminescens* and *X. stockiae*. The *E. coli* electrocompetent cells were prepared according to our established protocol (41). For *P. luminescens* and *X. stockiae*, overnight cultures containing the expression plasmids were diluted into 1.3-ml LB medium with appropriate antibiotics. The starting OD_600_ value was around 0.15. The fresh culture was grown at 30°C, 950 rpm for 4.5 h until the OD_600_ was around 0.85. After addition of the inducer L-(+)-arabinose to a final concentration of 2.5 mg/ml, the cells were grown at 30°C, 950 rpm for 30 min until the OD_600_ was around 1.15. Cells were then centrifuged for 30 s at 9500 rpm at 2°C. The supernatant was discarded, and the cell pellet was resuspended in 1-ml ice-cold GH buffer (10% glycerol, 2-mM HEPES, PH = 6.5) and centrifuged. The ice-cold GH buffer washing was repeated once more. After that, cells were resuspended in 30-μl ice-cold GH buffer and DNA was added. Two hundred nanogram PCR products were used in experiments of Figures 2D, 3C and 4C and E. One microgram of the PCR products was used for genome modification of *P. luminescens* and *X. stockiae*, to insert the inducible promoter in front of the gene clusters (Figure 5). Electroporation was performed using ice-cold cuvettes and an Eppendorf 2510 electroporator set at 1200 V. One milliliter LB medium was added after electroporation. The cells were incubated at 30°C for 100 min with shaking then spread on appropriate antibiotic plates.

**Figure 1.**
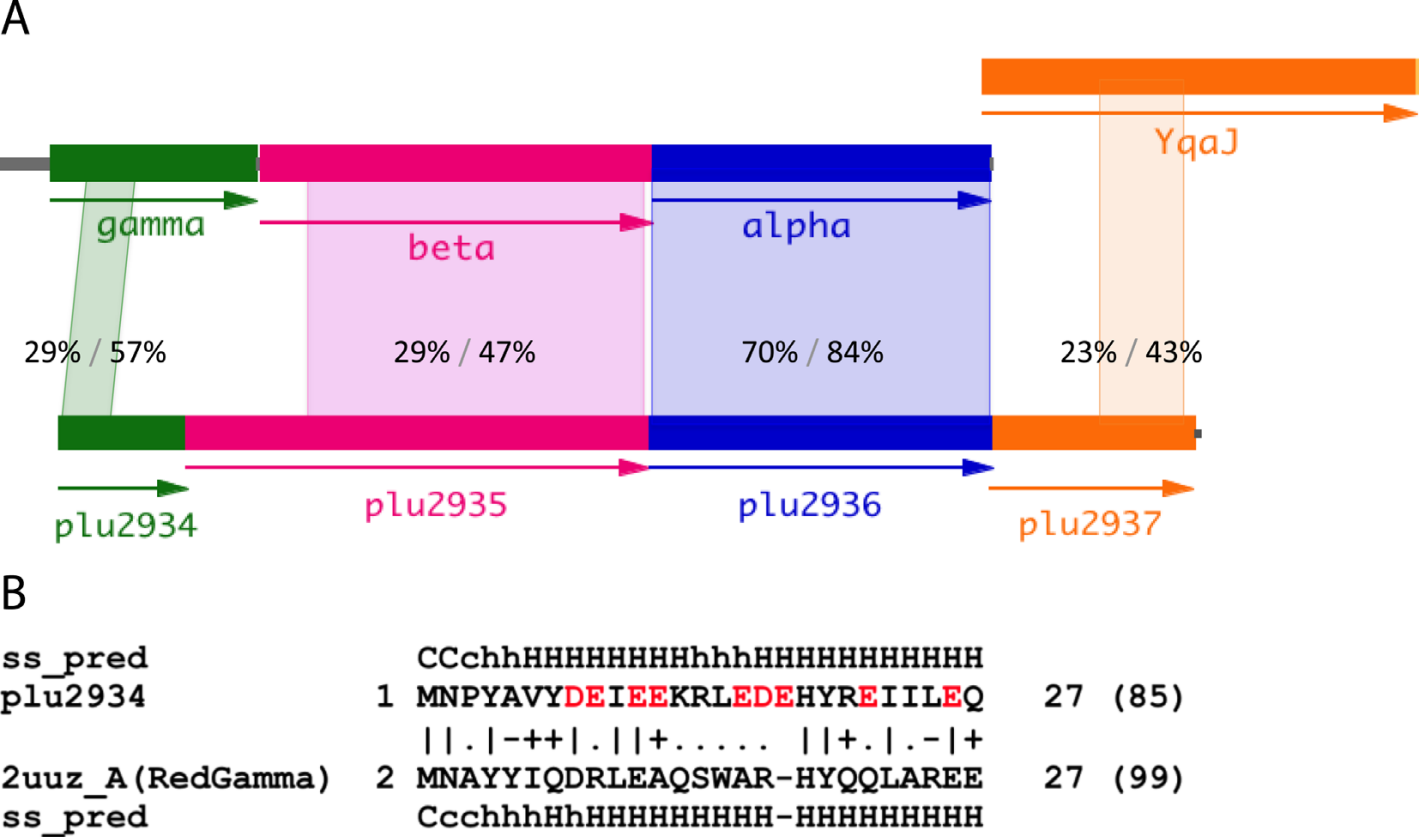
Bioinformatics analysis of Plu2934-6. (**A**) Operon architecture of λ Red and Plu2934-Plu2937. *Photorhabdus* prophage Plu2934, Plu2935 and Plu 2936 are related to Redγ (gamma), Redβ (beta) and Redα (alpha), respectively, encoded by λ phage. Plu2937 shares similarity with YqaJ encoded by the *Bacillus subtilis* prophage. The amino acid sequences were compared with NCBI protein Blast analysis. The extent, position, percentage identity and similarity are displayed between the homology regions. (**B**) Plu2934 protein homology detection of known structures using the HHpred interactive server. The Redγ structure is taken from PDB entry 2uuz chain A and exhibits alpha-helical structure. The negatively charged residues aspartic acid (D) and glutamic acid (E) are marked in red. ss_pred: secondary structure prediction by PSIPRED; H/h: alpha-helix; C/c: coli; upper case: high probability; lower case: low probability; quality of column match (bad: −; neutral:.; good +; very good |).

**Figure 2.**
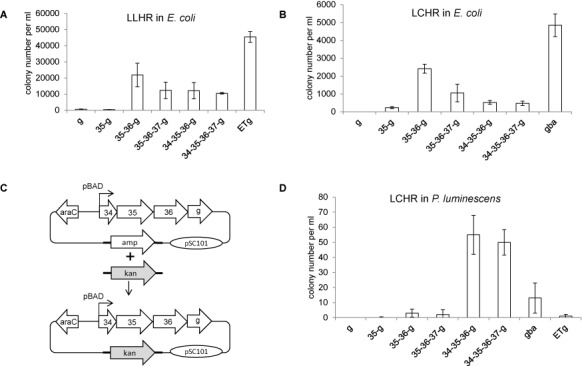
Recombineering with different protein combinations in *E. coli* and *P. luminescens*. (**A**) Results from a linear plus linear homologous recombination (LLHR) assay in *E. coli* (23) upon expression of Redγ (g); Plu2935 and Redγ (35-g), Plu2935/Plu2936 and Redγ(35–36-g), Plu2935/Plu2936/Plu2937 and Redγ (35-36-37-g), Plu2934/Plu2935/Plu2936 and Redγ (34-35-36-g), Plu2934/Plu2935/Plu2936/Plu2937 and Redγ (34-35-36-37-g) and full length RecE, RecT and Redγ (ETg). Expression was driven by the pBAD promoter in the pSC101-amp plasmid. (**B**) As in (A) except using a linear plus circular homologous recombination (LCHR) assay (23) and Redγ, Redβ and Redα (gba) was used as a reference instead of ETg. (**C**) Diagram of the LCHR assay used in *P. luminescens*. A PCR product carrying the kanamycin resistance gene (kan) flanked by 50-bp homology arms (represented by the thick lines) was integrated into the expression plasmid in place of the ampicillin resistance gene. (**D**) Results from the LCHR assay depicted in (C) upon expression of various protein combinations as defined in (A) and (B). Colonies were selected on kanamycin plates and counted. Error bars, SD; *n* = 3.

**Figure 3.**
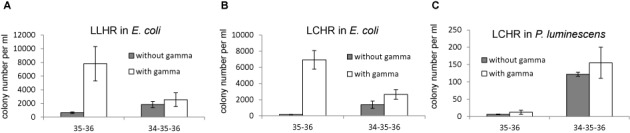
Functional analysis of Plu2934 and Redγ. (**A**) Result from the LLHR assay in *E. coli* comparing expression of Plu2935/Plu2936 (35–36) and Plu2934/Plu2936/Plu1936 (34-35-36) in the presence and absence of Redγ. **(B)** As in (A) except using the LCHR assay in *E. coli*. (**C**) As in (B) except using the LCHR assay of Figure 2C in *P. luminescens*. Colonies were selected on kanamycin plates and counted. Error bars, SD; *n* = 3.

**Figure 4.**
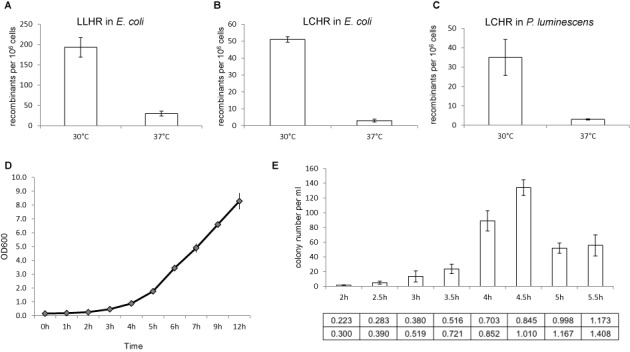
Optimization of recombineering in *P. luminescens*. (**A**) Results from the LLHR assay in *E. coli* mediated by Plu2934/Plu2935/Plu2936 evaluated at either 30 or 37°C. (**B**) Results from the LCHR assay in *E. coli* mediated by Plu2934/Plu2935/Plu2936 evaluated at either 30 or 37°C. (**C**) Results from the LCHR assay of Figure 2C in *P. luminescens* mediated by Plu2934/Plu2935/Plu2936 evaluated at either 30 or 37°C. Colonies were selected on kanamycin plates and counted. Error bars, SD; *n* = 3. (**D**) Growth curve of *P. luminescens* harboring the expression plasmid pSC101-BAD-34-35-36-amp. The optical density at 600 nm (OD_600_) was measured per hour from a starting OD_600_ of 0.15. (**E**) Results for the LCHR assay of Figure 2C in *P. luminescens* mediated by Plu2934/Plu2935/Plu2936. The starting OD_600_ of the culture was 0.15 and arabinose induction began at 2 h and then at 30-min intervals up to 5.5 h. The cultures were induced for 30 min harvesting to make cells for electroporation. The OD_600_ values before and after induction are listed below the time point. Colonies were selected on kanamycin plates and counted. Error bars, SD; *n* = 3.

**Figure 5.**
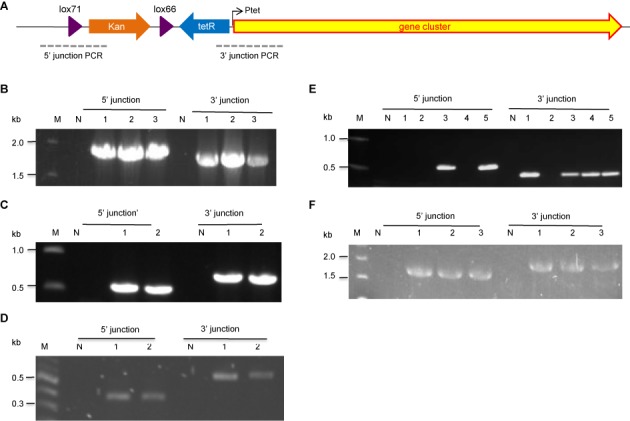
PCR verification of the proper insertion of the Ptet promoter by recombineering in *P. luminescens* and *X. stockiae*. (**A**) Schematic presentation of the PCR setup. (**B**) Insertion of P_tet_ promoter for *plu3263* expression in *P. luminescens*. Lane M is the NEB 1KB ladder. Lane N is the wild-type strain as negative control. Lanes 1–3 are recombinants. All three clones are correct. (**C**) Insertion of P_tet_ promoter for *plu1210-1222* expression in *P. luminescens*. Both clones 1 and 2 are correct. (**D**) Insertion of P_tet_ promoter for *Xe253* expression in *X. stockiae*. Both clones 1 and 2 are correct. (**E**) Insertion of P_tet_ promoter for *plu3123* expression in *P. luminescens*. Clones 3 and 5 are correct. (**F**) Insertion of P_tet_ promoter for *plu2670* expression in *P. luminescens*. All three clones are correct.

### Bioinformatic analysis

The *P. luminescens* genome was examined for Red/RecET phage protein homologs in the HHpred database pdb70_14Dec13 (derived from PDB on December 14, 2013) using the HHpred tool (http://toolkit.tuebingen.mpg.de/hhpred) (42). The secondary structure of the Plu2934 protein was predicted with Quick2D (http://toolkit.tuebingen.mpg.de/quick2_d) in the bioinformatics toolkit (http://toolkit.tuebingen.mpg.de/) (43). Position-Specific Iterative BLAST (PSI-BLAST) was used for homology searching in the database of non-redundant protein sequences (44). The catalytic domains of *plu* genes were annotated and the NRPS module substrates were predicted using antiSMASH analysis (45).

### Extraction and analysis of the compound from *P. luminescens* or *X. stockiae*

The recombineered strains were incubated in 100-ml flasks containing 30-ml LB medium with kanamycin (10 μg ml^−1^) or gentamicin (5 μg ml^−1^). The medium was inoculated with overnight culture (2%) and incubated for 3 h at 30°C on a rotary shaker. After induction with anhydrotetracycline (AHT) at a final working concentration of 0.2 μg ml^−1^, incubation was continued for another 3 days, and then 2% of absorber resin Amberlite XAD-16 was added and incubated overnight. The biomass and XAD-16 were harvested by centrifugation, and the resulting pellets were then extracted with methanol. The extracts were air-dried and dissolved in methanol for HPLC-MS analysis. The HPLC-MS measurements were performed on a Dionex Ultimate 3000 LC system using a BEHC-18, 50 × 2 mm, 1.7 μm d_p_ column (Waters, Germany). Separation of a 2-μl sample was achieved by a linear gradient with A (water + 0.1% formic acid) to B (acetonitrile + 0.1% formic acid) with a flow rate of 600 μl/min at 45°C. The gradient was initiated by a 0.5-min isocratic step at 5% B, followed by an increase to 95% B in 9 min then finally a 1.5-min step at 95% B before re-equilibration with initial conditions. UV spectra were recorded by a Diode Array Detectors (DAD) in the range of 200–600 nm. The mass spectrometry (MS) measurement was carried on an amaZon speed mass spectrometer (Bruker Daltonics, Bremen, Germany) using the Apollo ESI source. Mass spectra were acquired in centroid mode ranging from 200 to 2000 m/z in positive ionization mode using auto-MS^2^.

High-resolution mass spectrometry was performed on a Dionex Ultimate 3000 RSLC system using a BEH C-18, 100 × 2.1 mm, 1.7-μm d_p_ column (Waters, Germany) by injecting 2-μl methanolic extract. Separation was achieved by a linear gradient with A (water + 0.1% formic acid) to B (acetonitrile + 0.1% formic acid) at a flow rate of 550 μl/min at 45°C. The gradient was initiated by a 0.39-min isocratic step at 5% B, followed by an increase to 95% B in 18 min to end up with a 1.5-min flush step at 95% B before re-equilibration with initial conditions. UV spectra were recorded by a DAD in the range from 200 to 600 nm. Coupling the HPLC to the MS was supported by an Advion Triversa Nanomate nano-ESI system attached to a Thermo Fisher Orbitrap. Mass spectra were acquired in centroid mode ranging from 200 to 2000 m/z at a resolution of *R* = 30 000. The flow was split to 500 nl/min before entering the ion source.

### Feeding studies

Feeding experiments were performed in 5-ml LB medium containing 2.5 mg of commercially available L-valine-d_8_ (Deutero GmbH), L-leucine-d_3_ (Deutero GmbH), L-threonine-^13^C_4_, ^15^N (Isotec), respectively. Precursor stocks were dissolved in LB medium and sterile filtered.

## RESULTS

### Bioinformatic analysis of host-specific phage proteins in *P. luminescens*

Preliminary data in our laboratory indicated that the *lambda* Red proteins do not mediate efficient editing of the *P. luminescens* genome again suggesting host-specific interactions (25,29,30,46). Consequently we examined the Plu2934-Plu2940 operon in the *P. luminescens* genome sequence (37,46). This operon encodes seven proteins including a candidate exonuclease, Plu2936, which is 70% identical to Redα over its 226 amino acid sequence and is adjacent to a candidate SSAP, Plu2935 which shows significant similarity to Redβ (sequence identity 29% and similarity 47% in a 224 amino acid region). The next coding region, Plu2937, is annotated as a YqaJ-like viral recombinase domain. Plu2937 and *Bacillus subtilis* prophage YqaJ protein share a 44 amino acid region of 23% identity and 43% positive (Figure 1A). YqaJ is one of the three protein subunits that form a toroid, which is similar in structure to Redα and RecE exonucleases (47,48). Consequently, Plu2937 may also have a role in recombination. Plu2934, Plu2938, Plu2939 and Plu2940 had not been previously annotated. We searched using HHpred for candidate matches to known structures in the PDB database and found no suggestive matches for Plu2938, Plu2939 and Plu2940. However the 27 N-terminal residues of Plu2934 scored a best hit with the N-terminal helix H1 of *lambda* Redγ (HHpred Probab = 71.70 and E-value = 2.2) (Figure 1B). The percentage identity and similarity between Redγ and Plu2934 are 29% and 57%, respectively, in this 27 amino acid sequence (Figure 1A). Secondary structure predictions of these 27 residues using Quick2D (42) strongly indicated a helical structure. The Redγ N-terminal helix H1 has been proposed to mimic DNA using negatively charged residues that bind to the 3′ and 5′ channels of the RecBCD complex (21). Aspartates and glutamates are also prominent in this region of Plu2934 (Figure 1B). Furthermore *P. luminescens* appears to encode a RecBCD complex encoded by Plu0632, Plu0630 and Plu0633 with over 55% sequence identity (Supplementary Table S4). These data suggest that Plu2934 may act like Redγ, which mimics the shape and charge of duplex DNA, to inhibit the host exonuclease complex Plu0632/Plu0630/Plu0633.

### Functional dissection of Plu2934-Plu2937

Based on the bioinformatic analysis we omitted Plu2938-Plu2940 from the investigation. To evaluate various protein combinations of Plu2934/Plu2935/Plu2936/Plu2937 in *E. coli*, we first included *lambda* Redγ because it enhances homologous recombination mediated not only by Redαβ but also by the heterologous RecET system (1,3,23). Standard linear plus linear homologous recombination (LLHR) and linear plus circular homologous recombination (LCHR) assays were employed using a 2-kb linear dsDNA substrate with 50-bp homology arms to recombine with either a linear or a circular p15A-cm vector (23). In LLHR experiments, the combination of full length RecE, RecT and Redγ (ETg) was used as a positive reference and Redγ alone (g) was used as a negative control (Figure 2A). In LCHR experiments, the Red operon (gba) was used as a positive reference (Figure 2B). In both LLHR and LCHR assays, the candidate SSAP Plu2935 with Redγ (35g) did not mediate much homologous recombination. When its candidate partner exonuclease Plu2936 was included (35–36g), the efficiency was significantly enhanced. Addition of Plu2934, Plu2937 or both reduced efficiencies in both assays.

We then compared the efficiencies of the different combinations in *P. luminescens* using an LCHR assay based on integration of a kanamycin resistance gene with 50-bp homology arms into the pSC101 expression plasmid (Figure 2C). Both Red (gba) and RecET (ETg) were compared to the Plu proteins (Figure 2D). The results were dramatically different to those obtained in *E. coli*. The two Plu protein combinations with Redγ (34-35-36-g and 34-35-36-37-g) were much more efficient than gba, ETg and any other Plu protein combinations. Notably Plu2934 made a significant contribution, whereas Plu2937 had no apparent effect.

Because of the extremely low transformation efficiency of *P. luminescens*, the Plu system worked about 50 folds less efficiently in its natural host than in *E. coli* (Supplementary Figure S2).

### *In vivo* analysis of Plu2934 and Redγ

Based on the bioinformatic clues (Figure 1) we examined the function of Plu2934 by comparing Plu2935/Plu2936 to Plu2934/Plu2935/Plu2936 with or without Redγ in *E. coli* (Figure 3AB). In the absence of Plu2934, Redγ enhanced homologous recombination mediated by Plu2935 and Plu2936. In the absence of Redγ, Plu2934 increased efficiencies. However when both Plu2934 and Redγ were expressed, a 3-fold decrease compared to only Redγ was observed. Possibly Plu2934 and Redγ compete for binding to *E. coli* RecBCD and Redγ is a more effective inhibitor.

The same protein combinations were compared in *P. luminescens* using the LCHR assay (Figure 3C). Notably, Redγ had little effect on recombination, whereas Plu2934 dramatically enhanced it and co-expression of Plu2934 and Redγ did not have a negative effect. This indicates that Redγ is not active in *P. luminescens* and Plu2934 shows high activity in its endogenous host.

### Optimization of recombineering in *P. luminescens*

The optimal cultivation temperature of *P. luminescens* is 30°C and presumably its phage proteins are most active at this temperature. We examined the Plu2934/Plu2935/Plu2936 temperature optima by recombineering assays in *E. coli* and *P. luminescens* (Figure 4). For both LLHR and LCHR, as well as in both hosts, more recombination mediated by Plu2934/Plu2935/Plu2936 was observed at 30°C than 37°C.

Recombineering in *E. coli* is optimal when phage protein expression is induced at the beginning of log phase growth (OD_600_∼0.30–0.35) for ∼40 min at 37°C, which is about two cell divisions to OD_600_ ∼0.70–0.80 (23,41). To optimize a protocol for *P. luminescens*, we first plotted growth of the host carrying pSC101-BAD-34-35-36-amp at 30°C (Figure 4D). Under these conditions, the log phase started after 4.5 h. Based on the plotted growth curve, we then checked induction time windows from 2 to 5.5 h (Figure 4E). The optimal time for arabinose addition was 4.5 h, at which point the OD_600_ was 0.845. After a further 30 min of induction at 30°C, the OD_600_ had reached 1.010. Using the LCHR recombineering assay we found that electrocompetent cells prepared at this point yielded the most recombinants (Figure 4E). This work established our conditions for recombineering in *P. luminescens*. The same conditions were also suitable for *X. stockiae*, which has very similar growth properties.

### *In situ* activation of silent NRPS gene clusters

We developed this recombineering method so that we could prospect for secondary metabolites in the *P. luminescens* and *X. stockiae* genomes. Hence to validate the method we aimed to activate secondary metabolite pathways by inducible promoter insertion. First we tested the ability of the tetracycline-regulated promoter (P_tet_) to inducibly express green fluorescent protein (GFP) from pSC101 plasmids (Supplementary Figure S1). These plasmids were used as templates to amplify lox71-kanR-lox66-tetR-P_tet_ or lox71-gentaR-lox66-tetR-P_tet_ PCR products flanked by 75-bp homology arms to place the P_tet_ promoter cassette in front of *P. luminescens* gene clusters: *plu1210-1222, plu2670, plu3123* and *plu3263* and the *xe253* gene cluster of *X. stockiae*. Each electroporation yielded at least 20 kanamycin or gentamicin resistant colonies and most were correct according to colony PCR at both the 5′ and 3′ junctions (Figure 5).

**Figure 6.**
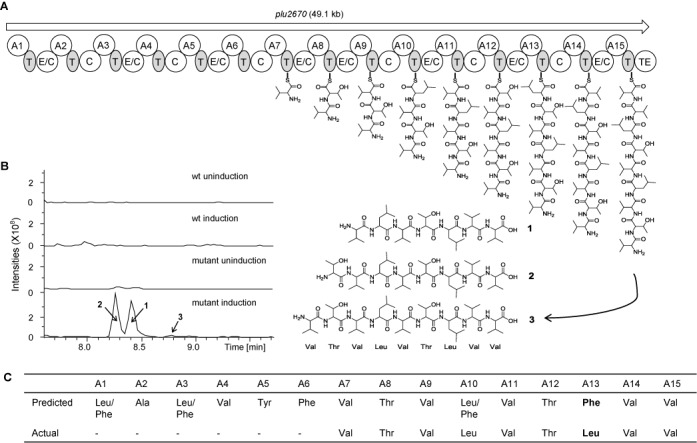
Induced expression of the NRPS *plu2670* gene cluster. (**A**) The structure of the *plu2670* gene cluster. A, adenylation; T, thiolation; TE, thioesterase; C, condensation; E/C, dual epimerization/condensation domain. Proposed biosynthetic pathway for compounds 1, 2 and 3 is illustrated. (**B**) HPLC-MS analysis (BPC 740–950+All MS) of methanol extracts of *P. luminescens* TT01 wild-type strain and mutant (*plu2670*-promoter) strain. The peaks (1, 2, 3) are marked in the chromatogram. (**C**) The predicted and actual substrates of each module are listed.

To further validate the engineering, we focused on the *plu2670* gene cluster, which is 49.1 kb and contains 15 NRPS modules (comprising A-T-E/C-A-T-C-A-T-E/C-A-T-C-A-T-E/C-A-T-C-A-T-E/C-A-T-E/C-A-T-C-A-T-E/C-A-T-C-A-T-E/C-A-T-C-A-T-E/C-A-T-TE) encoding the 15 amino acids nominated in Figure 6A based on antiSMASH prediction. Anhydrotetracyclin (AHT) induction produced three peaks: peak 1 (m/z 742.6 [M+H]^+^), peak 2 (m/z 843.6 [M+H]^+^) and peak 3 (m/z 942.7 [M+H]^+^) (Figure 6B). High-resolution mass spectrometry suggested that the elemental composition of peak 1 was C_36_H_68_N_7_O_9_ (Exact MS: 742.507, Delta ppm: 0.3), peak 2 was C_40_H_75_N_8_O_11_ (Exact MS: 843.554, Delta ppm: 0.7) and peak 3 was C_45_H_84_N_9_O_12_ (Exact MS: 942.623, Delta ppm: 5.5).

To further refine the structures, feeding studies (Supplementary Figure S3) were combined with detailed mass spectrometry fragment analysis (Supplementary Figure S4). Consequently we can propose a pathway and compounds corresponding to the three peaks (Figure 6A). The MS/MS analysis revealed that the six amino acids at the N-terminus were lost in all three compounds. For modules 7 to 15, the incorporated amino acid matched the *in silico* prediction except for module 13 where a leucine was found instead of the predicted phenylalanine (Figure 6C).

## DISCUSSION

Recombineering in *E. coli* was developed to extend DNA engineering technology beyond the limitations of conventional *in vitro* cut and paste methods using restriction enzymes, PCR and ligases. Beyond manipulations of recombinant DNA, recombineering provided precise and fluent access to the *E. coli* genome. Consequently the Red system has been applied to other prokaryotic genomes including *Salmonella, Yersinia, Agrobacterium* and *Shigella* (24–31). Prophage SSAPs RecTl from *Lactococcus* and Cpf0939 from *Clostridium* have been used for oligonucleotide-mediated site-directed mutagenesis (30,31). Endogenous exonuclease and SSAP pairs, which are capable of not only oligo repair but also cassette insertion, have been used in *P. syringae* (RecE_Psy_ and RecT_Psy_) (29) and *M. tuberculosis* (Gp60 and Gp61 from Che9c are homologs of RecE and RecT) (27). However apparent host-specific aspects have limited its wider application.

Here we describe a recombineering system based on a presumptive *Photorhabdus* prophage operon that is similar to *lambda* Red. Both operons have the same 5′ to 3′ arrangement encoding a presumptive host exonuclease inhibitor, an SSAP and a 5′-3′ dsDNA exonuclease (Figure 1A). The high degree of sequence conservation indicates that the two systems are derived from the same genetic ancestor. We therefore now rename Plu2934, Plu2935 and Plu2936 as Pluγ, Pluβ and Pluα, respectively. A summary of phage proteins used for recombineering in various species is presented in Supplementary Table S5.

Pluγ worked not only in its endogenous host but also in *E. coli* albeit at reduced efficiency. The observations that Pluγ works in a heterologous species and the hybrid system Pluαβ-Redγ works in *E. coli* imply that other hybrid systems may be usefully deployed. For example, enhancing the efficiency of RecE_Psy_/RecT_Psy_ or Gp60/Gp61 by adding Redγ or Pluγ could be fruitful. Pluγ is the first functionally identified Redγ homolog and its apparent 30°C temperature optima may extend its usefulness.

Several approaches to bioprospecting for secondary metabolites have been reported. Bicornutin, HeptaCycloTetraArginine-peptide (HCTA-peptide) A and HCTA-peptide B were identified in *Xenorhabdus* by straightforward screening for bioactive compounds (49,50). This approach is restricted to compounds that are expressed in hosts that can be cultivated. A different approach involves the use of heterologous hosts for expression. The *plu1881-1877* and *plu3263* silent gene clusters were directly cloned and transferred into a heterologous host to achieve expression. Consequently the novel compounds, Luminmycin A, Luminmide A and Luminmide B, were identified (23). Direct cloning and heterologous expression are effective approaches, especially for silent gene clusters identified in host bacterium that are difficult to culture or manipulate genetically. However, heterologous expression is often problematic. Firstly some heterologous hosts lack one or more of the enzymatic steps by which the various and/or complete metabolites are synthesized. To address this problem, the heterologous host can be modified to include the missing enzyme(s). For example, methylmalonyl-CoA production was introduced into *Pseudomonas putida* to facilitate polyketide synthase production (51). Secondly, the biosynthetic pathway may produce an intermediate or compound that is toxic to the heterologous host. For example, the isoprenoid precursor, isopentenyl pyrophosphate (IPP), is toxic to the heterologous host, *E. coli* (52). For another example, we observed that *plu1210-1222* and *plu3123* gene clusters could not be cloned in *E. coli* without 5′ mutations that ablated expression suggesting that the secondary metabolites were toxic (23).

Here we used *in situ* genome manipulation to activate secondary metabolite pathways. Most secondary metabolite pathways are silent under normal culture conditions. The lack of transcription can be circumvented by replacing the promoter if the genome can be engineered. This approach also avoids the complications involved with expression in heterologous hosts because the endogenous host should have all the required enzymology. Previously, insertion of an active promoter using a suicide plasmid carrying lengthy homology activated the *plu2180-plu2185* cluster and produced indigoidine (38). Here we advanced this approach by developing a Red-like recombineering system for *Photorhabdus* and *Xenorhabdus* and utilizing an inducible promoter. As previously established in *E. coli*, a recombineering approach has many advantages over reliance on endogenous homologous recombination. Notably, efficient recombination with shorter homology arms circumvents the lengthy construction times to make dedicated suicide plasmids. Additionally the use of Redγ-type exonuclease inhibitors greatly promotes recombination frequencies. Recombineering with Pluγβα presents fluency for bioprospecting in *Photorhabdus, Xenorhabdus* and closely related hosts (53). By analogy to the impact that recombineering has had on *E. coli*, our work is likely to have similar potential for functional genomics in these hosts. Further work to identify new Red-like or RecET-like operons in more distantly related hosts may further extend our capacities to precisely engineer genomes for bioprospecting and functional genomics.

## SUPPLEMENTARY DATA

Supplementary Data are available at NAR Online.

SUPPLEMENTARY DATA
