# Supplementary material for: A new recombineering system for Photorhabdus and Xenorhabdus
Source: Nucleic Acids Res. 2014 Dec 24;43(6):e36. doi: 10.1093/nar/gku1336 (PMC4381043; doi:10.1093/nar/gku1336)
Supplement: SUPPLEMENTARY DATA [file supp_43_6_e36__index.html]

A new recombineering system for Photorhabdus and Xenorhabdus — SUPPLEMENTARY DATA 

# A new recombineering system for *Photorhabdus* and *Xenorhabdus*

## SUPPLEMENTARY DATA

**Files in this Data Supplement:**

- SUPPLEMENTARY DATA
