# Supplementary material for: A new recombineering system for Photorhabdus and Xenorhabdus
Source: Nucleic Acids Res. 2014 Dec 24;43(6):e36. doi: 10.1093/nar/gku1336 (PMC4381043; doi:10.1093/nar/gku1336)
Supplement: SUPPLEMENTARY DATA [file supp_gku1336_Jia_Plu_supplementary_gku1336.pdf]

**Table S1. Bacterium strains of *Escherichia coli*, *Photorhabdus luninescens* and *Xenorhabdus stockiae***

| Strain | Genome type | References or sources |
|---|---|---|
| GB2005 | F-*mcr*A Δ(*mrr-hsd*RMS-*mcr*BC) φ80*lac*ZΔM15 Δ*lac*X74 *rec*A1 *end*A1 *ara*D139 Δ(ara, leu)7697 *gal*U *gal*K λ *rps*L *nup*G *fhu*A::IS2 *rec*ET phage T1-resistent (*E. coli*) | (1) |
| GB05-dir | GB2005, araC-BAD-ETγA (*E. coli*) | (2) |
| GB08-red | GB2005, araC-BAD-γβαA, ΔlacZ (*E. coli*) | (1) |
| *P. luminescens* TT01 | Wild type strain of *Photorhabdus luminescens* | (3) |
| 1210-promoter | $P_{tet}$ insertion for expression of gene cluster plu1210-1222 in *P. luminescens*, KmR | This study |
| 3123-promoter | $P_{tet}$ insertion for expression of gene cluster plu3123 in *P. luminescens*, KmR | This study |
| 3263-promoter | $P_{tet}$ insertion for expression of gene cluster plu3263 in *P. luminescens*, GentaR | This study |
| 2670-promoter | $P_{tet}$ insertion for expression of gene cluster plu2670 in *P. luminescens*, GentaR | This study |
| *X. stockiae* | Wild type strain of *Xenorhabdus stockiae* | Hunan, China |
| 253-promoter | $P_{tet}$ insertion for expression of gene cluster Xe253 in *X. stockiae*, KmR | This study |

## Table S2. Expression plasmids and PCR template

| Plasmid | Characteristics | References or sources |
|---|---|---|
| pSC101-BAD-g-amp | *redγ* under BAD promoter | (2) |
| pSC101-BAD-35-g-amp | *plu2935* and *redγ* under BAD promoter | This study |
| pSC101-BAD-35-36-g-amp | *plu2935/plu2936* and *redγ* under BAD promoter | This study |
| pSC101-BAD-35-36-amp | *plu2935/plu2936* under BAD promoter | This study |
| pSC101-BAD-35-36-37-g-amp | *plu2935/plu2936/plu2937* and *redγ* under BAD promoter | This study |
| pSC101-BAD-34-35-36-g-amp | *plu2934/plu2935/plu2936* and *redγ* under BAD promoter | This study |
| pSC101-BAD-34-35-36-amp | *plu2934/plu2935/plu2936* under BAD promoter | This study |
| pSC101-BAD-34-35-36-37-g-amp | *plu2934/plu2935/plu2936/plu2937* and *redγ* under BAD promoter | This study |
| pSC101-BAD-ETg-amp | *rec*ET and *redγ* under BAD promoter | (2) |
| pSC101-BAD-gba-amp | *Redγβα* under BAD promoter | (2) |
| pSC101-lox71-kanR-lox66-Ptet-GFP | GFP under $P_{tet}$, PCR templates to amplify lox71-kanR-lox66-Ptet (plasmid DNA digested with *Ava*I and *Bam*HI) | This study |
| pR6K-Tps-gentaR-tetR-T7RP | PCR templates to amplify lox71- gentaR-lox66-Ptet | This study |
| P15A-cm | A 2 kb plasmid with 15A rorigin of replication and chloramphenicol resistence marker | (2) |

**Table S3. Oligonucleotides**

| Gene | Primers | 5' - 3' | Application |
|---|---|---|---|
| *plu2935* | ex plu2935-5<br>ex plu2935-3 | TCGCAACTCTCTACTGTTTCTCCATACCCGTTTTTTTGGGCTAGCAGGAGGAACAGCTGATGAGCACAGCAGTACAAAA<br>GGGGTTAGTGAATGCTTTTGCTTGATCTCAGTTTCAGTATTAATATCCATGGTGAATTCCTCCTAGATCTTTATGATGCCTTTTTCCTTA | Expression plasmid |
| *plu2935-2936* | ex-plu2935-2936-5<br>ex plu2935-2936-3 | TCACCCGCCGAATTAACAGA<br>GCTTGATCTCAGTTTCAGTATTAATATCCATGGTGAATTCCTCCTAGATCTTCATTTCCATTGATCGCCAA | Expression plasmid |
| *plu2934-2936* | plu34-36-amp-5<br>plu34-36-amp-3 | ACGAATCATTAAATGAAATCGGCTTCAAATTTGGCGATCAATGGAAATGACAGCTGTCAGAAGAACTCGTCAAGAA<br>CTTGATCTCAGTTTCAGTATTAATATCCATGGTGAATTCCTCCTGATATCAGCTGGCTTGCAGTGGGCTTACAT | Expression plasmid |
| *plu2935-2937* | plu35-37-amp-5<br>plu35-37-amp-3 | CAACTCTCTACTGTTTCTCCATACCCGTTTTTTTGGGCTAGCAGGAGGAACAGCTGTCAGAAGAACTCGTCAAGAA<br>GTCTTGAGCGGGTTGATGATTTCATAAACTTTTTGTACTGCTGTGCTCATCAGCTGCTTGCAGTGGGCTTACAT | Expression plasmid |
| *plu2934-2937* | pluT-5<br>pluT-3 | CTACTGTTTCTCCATACCCGTTTTTTTGGGCTAGCAGGAGGAATTCACTATGAACCCATATGCAGTTTATGAT<br>GGGGTTAGTGAATGCTTTTGCTTGATCTCAGTTTCAGTATTAATATCCATGGTGAATTCCTCCTGATATCTCACCTCACTTAAACTGATTG | Expression plasmid |
| kanR gene | Tem-km-5<br>Tem-km-3 | GCGGTAGTTTATCACATACCGTTCGTATAATGTATGCTATACGAAGTTATGCTTGCAGTGGGCTTACAT<br>AATAAAAAAGGGGACCTCTAGGGTCCCCAATTAATTAGTAATATAATCTATCAGAAGAACTCGTCAAGAAG | PCR template |
| GFP | eGFP-5<br>eGFP-3 | GAGAAAAGTGAAATGAATAGTTCGACAAAAATCTAGCAGGAGGAATTCATATGACCATGATTACGCATCA<br>AGCGGTATCATCAACAGGCTTACCCGTCTTACTGTCTAGACTCGAGAAGCTTACTTGTACAGCTCGTCCA | PCR template |
| *plu1210* | 1210-5<br>1210-3 | TAACTTTGGAAAATGAATGACGTAGGCAGTTTAGCTAATCCATTTTAAATTAATGCGGTAGTTTATCACA<br>GAATACAGTGTGATCTCACTATTTGGAGAGCAAATCGTGTAAGGTAGCATATGAATTCCTCCTGCTAGAT | Insertion promoter |

| Gene | Primers | 5' - 3' | Application |
|---|---|---|---|
| *plu3123* | 3123-5 | ATTGGTGATGGTTGTCAGAAATGACAGTTTTCAAATTTCTAACAGATTCGTAATGCGGTAGTTTATCACA | Insertion promoter |
| | 3123-3 | AGGAGTTCAGCTTTAAAATCACTTCCTGCTTGAGCGATGCTATCTTTCATATGAATTCCTCCTGCTAGAT | |
| *xe253* | 253-5 | TGATTTTTTATATTTTAGAGAGAAAAAGTCAAAGAGAGAGGGATACGCGCTAATGCGGTAGTTTATCACA | Insertion promoter |
| | 253-3 | GGGAAACGGCAAGATATTCCCACAATAGCAATTTTGTCTCTGTCATGCATATGAATTCCTCCTGCTAGAT | |
| *plu2670* | PGenta2670-5 | CCGCTTGGGATAAGTGGGGCAAAAATAAAAAAACACTTGTGATGATTGCTAAGAATGATAGTTGCCAAATATCTAAGC TGAATTACATTCCCAACCG | Insertion promoter |
| | PGenta2670-3 | TATATTTTCTGATGTCTGAGAGAGAAGTTCGGCTTTAAGGATACTTTCCGCTTTTTTAGTAATGCTATCTTTCATTTAGT GCCTCTTCTCTATCACTGA | |
| *plu3263* | 3263PtetGen-5 | TCTTGGGGGGAAGGAAGAAGGGCAAAACATTTGTGATGACTGTTGAGAATGACAGTTTTCAAATAACTAACAGATAGC TGAATTACATTCCCAACCG | Insertion promoter |
| | 3263PtetGen-3 | ACCGACCTTGTTTGACAGTACTTGCACTAATCCAGCATCAAAGATAATTTCCTTTTTAGCCATGCTATCTTTCATTTAGT GCCTCTTCTCTATCACTGA | |

The homology arms are underlined.

**Table S4. Sequence alignment between *E. coli* RecBCD and Plu0632/Plu0630/Plu0633**

| | **Size (aa)** | **Identities** | **Positives** | **Gaps** |
|---|---|---|---|---|
| RecB<br>Plu0632 | 1180<br>1202 | 693/1185 (58%) | 889/1185 (75%) | 19/1185 (1%) |
| RecC<br>Plu0630 | 1122<br>1129 | 675/1131 (60%) | 852/1131 (75%) | 11/1131 (0%) |
| RecD<br>Plu0633 | 608<br>618 | 344/609 (56%) | 427/609 (70%) | 10/609 (1%) |

Results from NCBI blastp (protein-protein BLAST) using default parameters.

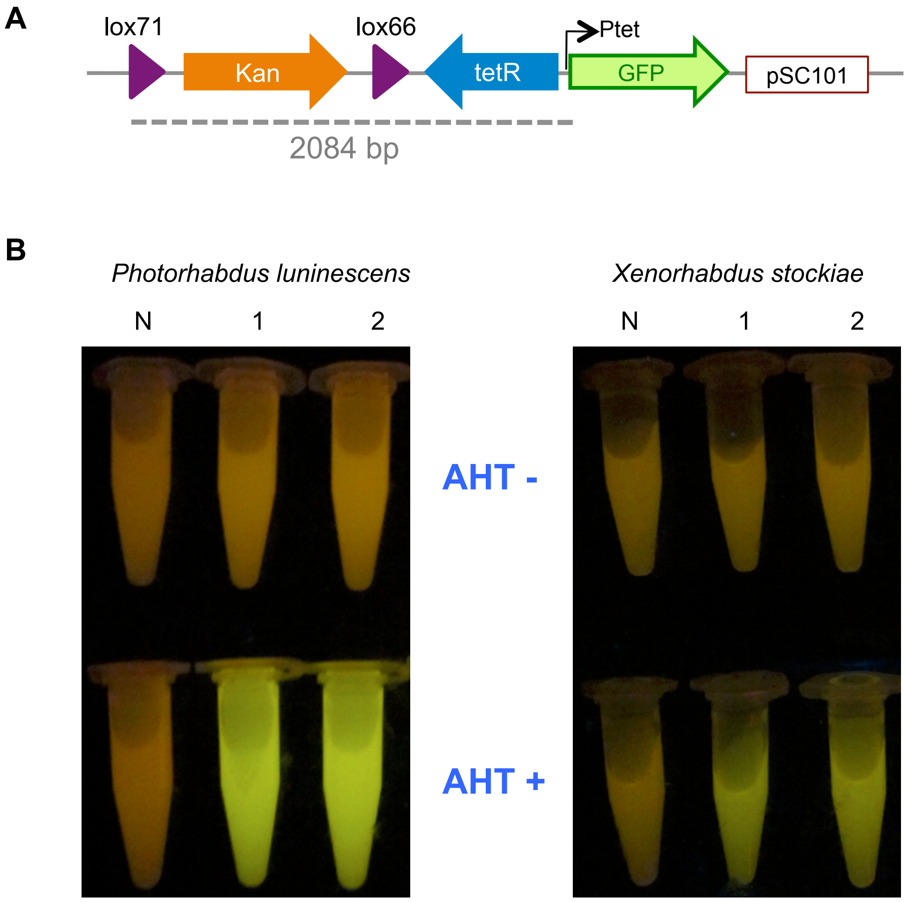


**Figure S1. Functional test of the $P_{tet}$ inducible promoter in *P. luminescens* and *X. stockiae*.** (**A**) Schematic presentation of GFP reporter under the $P_{tet}$ promoter in a pSC101 plasmid. The plasmid is for function test of the $P_{tet}$ promoter and PCR template of the 2 kb lox71-kanR-lox66-$P_{tet}$ cassette. (**B**) The pSC101-lox71-kanR-lox61-$P_{tet}$-GFP was transformed into *P. luminescens* and *X. stockiae* for function test. Negative controls were set up for both *P. luminescens* and *X. stockiae,* which are wild type strains without the GFP reporter plasmid (N). The number 1 and 2 were *P. luminescens* and *X. stockiae* harbouring the pSC101-lox71-kanR-lox66-$P_{tet}$-GFP plasmid, with addition of anhydrotetracycline (AHT+) or without addition of anhydrotetracycline (AHT-).

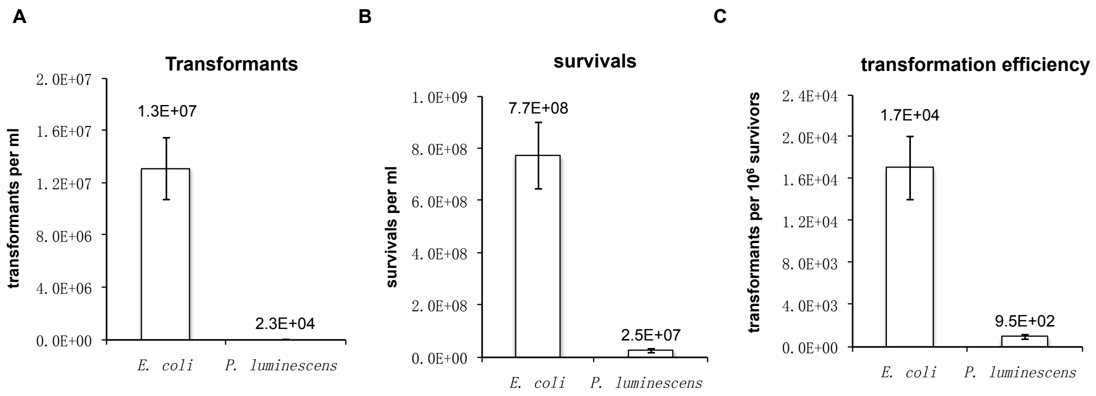


**Figure S2. Transformation efficiency test of *E. coli* and *P. luminescens*.** The pACYC177 plasmid (New England BioLabs) was used for transformation. The plasmid is a 3.9-kb ampicillin and kanamycin-resistant cloning vector with the p15A origin of replication. Fresh 1.4ml cultures were used to prepare electrocompetent cells and 200 ng of the plasmid DNA was introduced by electroporation. The cells were flushed out with 1 ml LB from the cuvette into a fresh tube to start the recovery for 1 hour. (**A**) The cultures were streaked on the LB agar plate with ampicillin and kanamycin and colony numbers were counted to determine transformants. (**B**) The cultures were streaked on LB agar plate without any antibiotic to determine total surviving colonies. (**C**) The transformation efficiency is presented as the ratio of transformants to survivors.

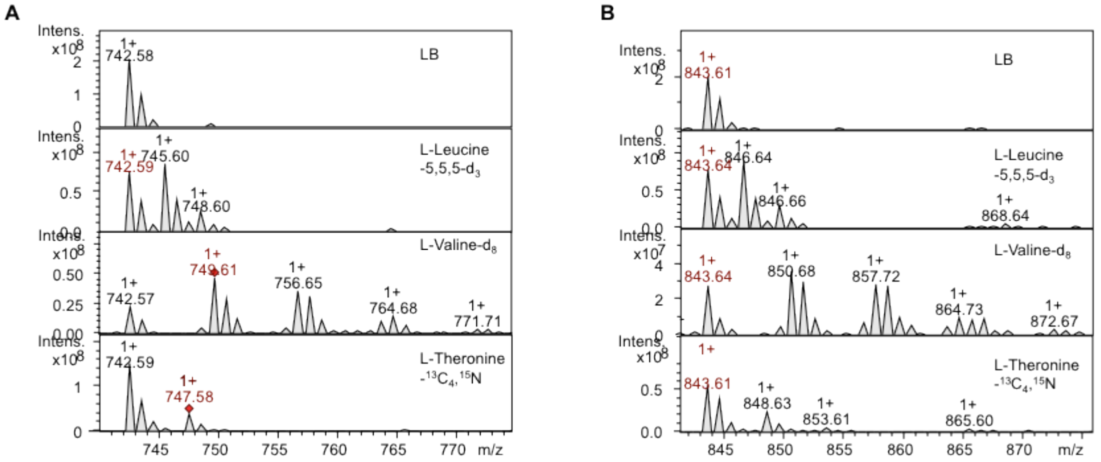


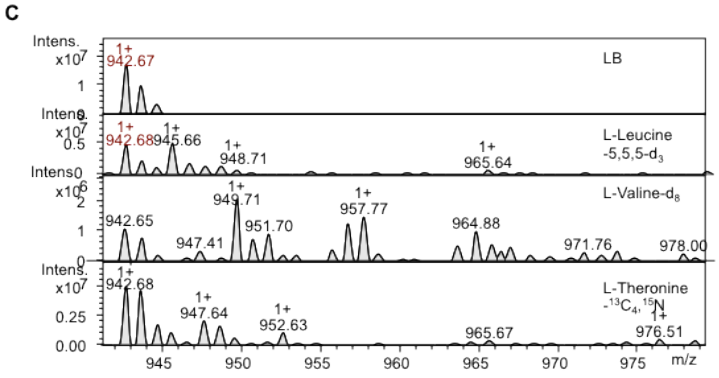


**D**

| | Peak 1 742.6 | Peak 2 843.6 | Peak 3 942.7 |
|---|---|---|---|
| **Leu** | 2 | 2 | 2 |
| **Val** | 4 | 4 | 5 |
| **Thr** | 1 | 2 | 2 |

**Figure S3. MS isotope patterns resulting from feeding isotope-labeled L-leucine, L-valine and L-threonine to *P. luminescens* mutant (*plu2670*-promoter) strain after anhydrotetracycline (AHT) induction.** Incorporation of labeled precursors is proven by characteristic mass shifts of the isotope patterns of (**A**) 742.58 m/z, (**B**) 843.61 m/z, and (**C**) 942.67 m/z. All feedings were performed in standard LB medium (LB) supplemented with 500 mg/L of labeled precursor. (**D**) Summary of the amino acids compositions for each compound based on the observed m/z shifts upon incorporation of labeled precursors.

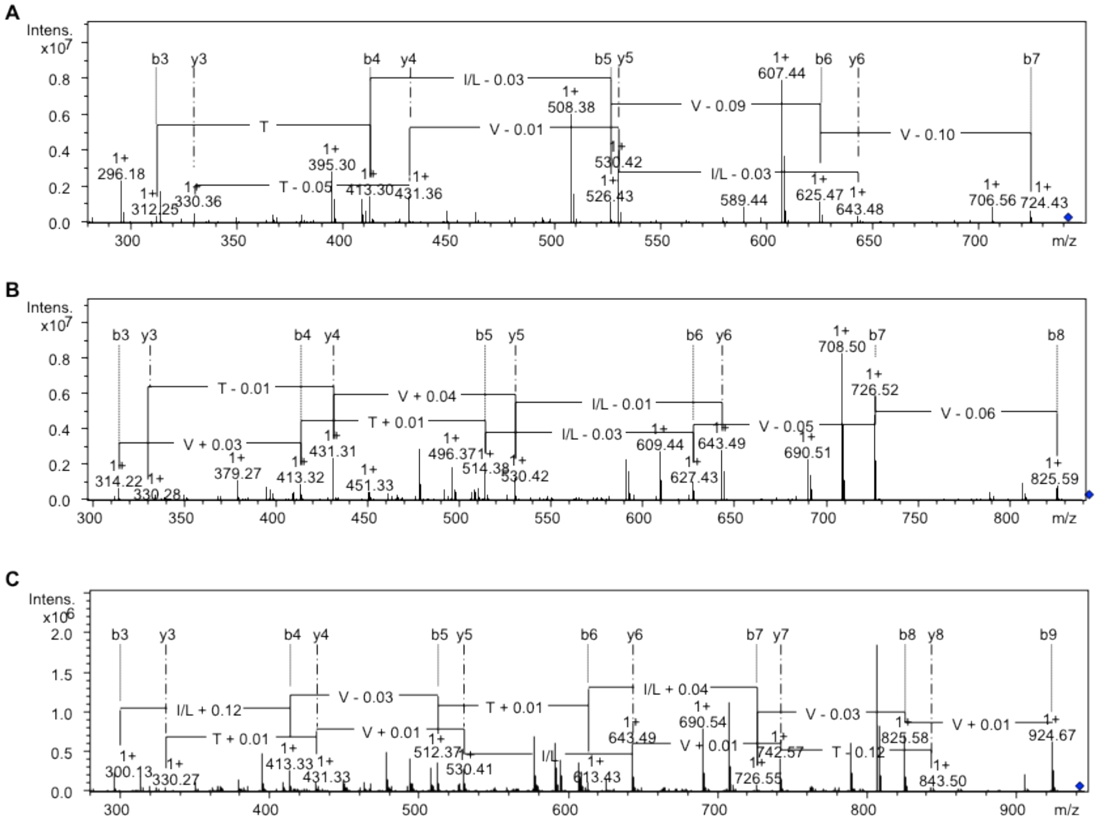


**Figure S4. MS/MS spectrum analysis of the three compounds.** CID-spectrum of 742.58 m/z peak 1 (**A**), 843.61 m/z peak 2 (**B**) and 942.67 m/z peak 3 (**C**). The y-ions are labeled with dashed/dotted lines and b-ions with dotted lines. Residues between adjacent $y_n$ and $b_n$-ions are labeled with the amino acid (one letter code) and the m/z deviation of this particular amino acid shift.

**Table S5. Phage proteins used in various bacteria for recombineering**

| Species | Classification | 5'-3' exonuclease | ssDNA annealing protein | Host exonulease inhibitor | Reference |
|---|---|---|---|---|---|
| *Escherichia coli* | Gram-negative | RecE (*E. coli*) | RecT (*E. coli*) | Redγ (phage λ) | (4) |
| *Escherichia coli* | Gram-negative | Redα (phage λ) | Redβ (phage λ) | Redγ (phage λ) | (5) |
| *Salmonella enterica* | Gram-negative | Redα (phage λ) | Redβ (phage λ) | Redγ (phage λ) | (6) |
| *Yersinia pseudotuberculosis* | Gram-negative | Redα (phage λ) | Redβ (phage λ) | Redγ (phage λ) | (7) |
| *Shigella* | Gram-negative | Redα (phage λ) | Redβ (phage λ) | Redγ (phage λ) | (8) |
| *Mycobacterium tuberculosis* | Gram-positive | Gp60 (phage *Che9c*) | Gp61 (phage *Che9c*) | - | (9) |
| *Pseudomonas syringae* | Gram-negative | $RecE_{Psy}$ (*P. syringae*) | $RecT_{Psy}$ (*P. syringae*) | - | (10) |
| *Lactococcus lactis* | Gram-positive | - | RecTl (*L. reuteri*) | - | (11) |
| *Lactococcus reuteri* | Gram-positive | - | RecTl (*L. reuteri*) | - | (11) |
| *Agrobacterium tumefaciens* | Gram-negative | Redα (phage λ) | Redβ (phage λ) | Redγ (phage λ) | (12) |
| *Clostridium acetobutylicum* | Gram-positive | - | Cpf0939 (*C. perfringens*) | - | (13) |
| *Photorhabdus luminescens* | Gram-negative | Pluα (*P. luminescens*) | Pluβ (*P. luminescens*) | Pluγ (*P. luminescens*) | This study |
| *Xenorhabdus stockiae* | Gram-negative | Pluα (*P. luminescens*) | Pluβ (*P. luminescens*) | Pluγ (*P. luminescens*) | This study |

## Reference:


1. Fu, J., Teucher, M., Anastassiadis, K., Skarnes, W., Stewart, A.F. (2010) A Recombineering Pipeline to Make Conditional Targeting Constructs. *Methods Enzymol.* , **477**, 125-144.
2. Fu, J., Bian, X., Hu, S., Wang, H., Huang, F., Seibert, P.M., Plaza, A., Xia, L., Müller, R., Stewart, A.F. *et al.* (2012) Full-length RecE enhances linear-linear homologous recombination and facilitates direct cloning for bioprospecting. *Nature Biotechnology*, **30**, 440-446.
3. Tang, Z., Zhang, Y., Stewart, A.F., Geng, M., Tang, X., Tu, Q. and Yin, Y. (2010) High-level expression, purification and antibacterial activity of bovine lactoferricin and lactoferrampin in *Photorhabdus luminescens*. *Protein Expression and Purification*, **73**, 132-139.
4. Zhang, Y., Buchholz, F., Muyrers, J.P.P. and Stewart, A.F. (1998) A new logic for DNA engineering using recombination in *Escherichia coli*. *Nature Genetics*, **20**, 123-128.
5. Zhang, Y., Muyrers, J.P.P., Testa, G. and Stewart, A.F. (2000) DNA cloning by homologous recombination in *Escherichia coli*. *Nature Biotechnology*, **18**, 1314-1317.
6. Bunny, K., Liu, J. and Roth, J. (2002) Phenotypes of *lex*A mutations in *Salmonella* enterica: evidence for a lethal *lex*A null phenotype due to the Fels-2 prophage. *Journal of bacteriology*, **184**, 6235-6249.
7. Derbise, A., Lesic, B., Dacheux, D., Ghigo, J.M. and Carniel, E. (2003) A rapid and simple method for inactivating chromosomal genes in *Yersinia*. *FEMS Immunology & Medical Microbiology*, **38**, 113-116.
8. Ranallo, R.T., Barnoy, S., Thakkar, S., Urick, T. and Venkatesan, M.M. (2006) Developing live *Shigella* vaccines using λ Red recombineering. *FEMS Immunology & Medical Microbiology*, **47**, 462-469.
9. Van Kessel, J.C. and Hatfull, G.F. (2006) Recombineering in *Mycobacterium tuberculosis*. *Nature Methods*, **4**, 147-152.

10. Swingle, B., Bao, Z., Markel, E., Chambers, A. and Cartinhour, S. (2010) Recombineering using RecTE from *Pseudomonas syringae*. *Applied and environmental microbiology*, **76**, 4960-4968.

11. van Pijkeren, J.-P. and Britton, R.A. (2012) High efficiency recombineering in *lactic acid* bacteria. *Nucleic acids research*, **40**, e76.

12. Hu, S., Fu, J., Huang, F., Ding, X., Stewart, A.F., Xia, L. and Zhang, Y. (2013) Genome engineering of *Agrobacterium tumefaciens* using the *lambda* Red recombination system. *Applied microbiology and biotechnology*, **98**, 1-8.

13. Dong, H., Tao, W., Gong, F., Li, Y. and Zhang, Y. (2013) A functional *rec*T gene for recombineering of *Clostridium*. *Journal of biotechnology*, **173**, 65-67.
